# Prognostic value of platelet count-related ratios on admission in patients with pyogenic liver abscess

**DOI:** 10.1186/s12879-022-07613-x

**Published:** 2022-07-21

**Authors:** Shixiao Li, Sufei Yu, Jiajia Qin, Minfei Peng, Jiao Qian, Peng Zhou

**Affiliations:** 1 Department of Clinical Microbiology Laboratory, Taizhou Hospital of Zhejiang Province Affiliated to Wenzhou Medical University, Taizhou, Zhejiang China; 2 Department of Pharmacy, Taizhou Hospital of Zhejiang Province Affiliated to Wenzhou Medical University, No. 150, Ximen Street, Taizhou, 317000 Zhejiang China

**Keywords:** Pyogenic liver abscess, C-reactive protein-to-platelet ratio (CPR), Neutrophil-to-lymphocyte*platelet ratio (NLPR), Fibrinogen-to-platelet ratio (FPR), Prognosis

## Abstract

**Objective:**

The purpose of the current study was to evaluate the association between C-reactive protein-to-platelet ratio (CPR), neutrophil-to-lymphocyte*platelet ratio (NLPR) and fibrinogen-to-platelet ratio (FPR) and the prognoses of pyogenic liver abscess (PLA) patients.

**Methods:**

A cohort of 372 patients with confirmed PLA were enrolled in this retrospective study between 2015 and 2021. Laboratory data were collected on admission within 24 h. The demographic characteristics and clinical features were recorded. Risk factors for outcomes of PLA patients were determined via multivariate logistic regression analyses, and optimal cut-off values were estimated by using the receiver operating characteristic (ROC) curve analysis.

**Results:**

Out of 372 patients, 57.8% were men, 80 (21.5%) developed sepsis, and 33 (8.9%) developed septic shock. The levels of CPR, NLPR and FPR were significantly increased in the development of sepsis, and prolonged hospital stays in PLA patients. The multivariate logistic regression analysis indicated that the CPR (OR: 2.262, 95% CI: 1.586–3.226, *p* < 0.001), NLPR (OR: 1.118, 95% CI: 1.070–1.167, *p* < 0.001) and FPR (OR: 1.197, 95% CI: 1.079–1.329, *p* = 0.001) were independent risks of PLA patients with sepsis, and NLPR (OR: 1.019, 95% CI: 1.004–1.046, *p* = 0.019) was shown to be an independent predictor of prolonged hospital stays. The ROC curve results showed that the three biomarkers had different predictive values, and CPR proved to work best, with a ROC value of 0.851 (95% CI: 0.807–0.896, *p* < 0.001) for sepsis.

**Conclusion:**

Higher levels of CPR, NLPR and FPR were associated with a higher risk of poor outcomes. Moreover, a high CPR level performed best when predicting the clinical outcome in PLA patients.

**Supplementary Information:**

The online version contains supplementary material available at 10.1186/s12879-022-07613-x.

## Introduction

Pyogenic liver abscess (PLA) is a serious yet relatively uncommon infectious disease with severe morbidity and mortality [1]. Its clinical incidence varies depending on the populations being studied, but the incidence has been reported to range from 1.07 to 3.59 per 100,000 hospital admissions in the West and up to 17.59 per 100,000 in the East [2]. Moreover, the mortality rate has a range of 2.8–10.8% [3]. Factors predisposing to PLA include advanced age, diabetes mellitus, liver cirrhosis and malignancy [4, 5]. PLA is caused by various bacteria. At present, *Klebsiella pneumonia* is the most frequently isolated bacterium of liver abscesses in Asia, followed by *Escherichia coli* [6, 7]. With the continuous improvement in diagnostic technology and treatment, the prognoses of PLA patients have become significantly improved. However, the complications of PLA are still a serious clinical problem, especially when combined with high risk factors or concomitant diseases. Therefore, it is necessary to screen for laboratory indices to develop convenient and easy-to-measure indicators of early predictions for the prognosis of PLA.

To date, numerous inflammation-related indices such as C-reactive protein (CRP), neutrophil-to-lymphocyte ratio (NLR) and fibrinogen (FIB), have been extensively used as prognostic values for many diseases [8–10]. Platelets play an important role in haemostasis and coagulation and are regulators of inflammation by controlling inflammation, and tissue integrity, as well as by defending against infection [11]. Some studies have indicated that patients with severe sepsis/septic shock have lower platelet count [12]. The prognostic ability of platelet count-related indices has been investigated in a variety of diseases, such as cancers, sepsis and liver cirrhosis [13–16]. The platelet count-related ratios may be a more promising biomarker of inflammation than conventional indicators. In our study, the combination of C-reactive protein, NLR and FIB with PLT indicated inflammation and coagulation status. Thus, we hypothesized that PLT-related ratios may provide potential early predictions of PLA patients. However, no study has compared the association between platelet count-related ratios and the prognostic value of pyogenic liver abscess. Therefore, the main aim of the present analysis was to explore the relationship between platelet-related ratios, including the C-reactive protein-to-platelet ratio (CPR), neutrophil-to-lymphocyte* platelet ratio (NLPR) and fibrinogen-to-platelet ratio (FPR), and the clinical characteristics and prognoses of PLA patients.

## Materials and methods

### Study population

This was a retrospective single-center survey performed at Taizhou Hospital of Zhejiang Province from January 2015 to June 2021. This study included 372 PLA patients (International Classification of Disease, Clinical Modification 572.0). PLA was defined as any abscess found within the liver parenchyma that was of bacterial origin [17]. All of the diagnoses of PLA were based on the typical clinical features, such as fever, chills and abdominal pain, combined with imaging examinations of the abscess cavity in the liver as diagnosed by liver ultrasonography and/or computerized tomography (CT), blood culture and/or pus from liver aspirates [18]. The inclusion criteria for the patients were as follows: (1) ≥ 18 years of age, (2) prior hospitalizations for PLA. The exclusion criteria for the patients were as follows: (1) patients who had an amebic liver abscess, fungal liver abscess or parasitic liver abscess (n = 2), (2) patients who were transferred to another hospital after admission (n = 3), (3) patients who developed PLA during hospitalization due to other conditions (n = 2), (4) patients who did not complete medical records (n = 31). Sepsis was diagnosed according to the third international consensus [19]. Prolonged hospital stay was defined as more than 21 days of hospital admission. All PLA patients were empirically treated with broad-spectrum antibiotics such as third-generation cephalosporins or β-lactams/β-lactamase inhibitors combined with metronidazole, or the antibiotics were adjusted based on antimicrobial susceptibility results. Four to six weeks were recommended for treatment with antibiotics alone. Patients with good initial drainage responses should have been treated with intravenous antibiotics for 2–4 weeks, whereas patients with incomplete drainage should have been treated with intravenous antibiotics for 4–6 weeks. According to the number and sizes of the abscesses, the degree of abscess liquefaction, antibiotics alone or antibiotics combined with percutaneous drainage or surgical drainage were selected. Percutaneous catheter drainage was performed under ultrasound or CT guidance if the diameter of the liver abscess was ≥ 3 cm. Patients who had ruptures of the abscesses with peritonitis, complications of other biliary tract diseases requiring surgery or unresponsiveness to the above treatments were selected for surgical drainage.

The study protocol complied with the principles of the Helsinki Declaration and was approved by the Institutional Medical Ethics Committee of Taizhou Hospital of Zhejiang Province. The informed consent requirement was waived due to the retrospective study design.

### Data collection

Data were obtained from the hospital medical electronic records system. For each enrolled patient, the following clinical data were documented: demographic data, clinical symptoms, underlying conditions, etiologies, surgery history, laboratory results, imaging findings, therapeutic strategies and outcomes. The fasting venous blood of patients was extracted within the first 24 h after hospital admission. The complete blood count analysis was conducted on BC-6800 plus automatic blood cell counter (Mindray, China). The blood biochemical index was detected by using AU5800 (Beckman Coulter, USA) automatic biochemical analyser. C-reactive protein was quantified by using Immage 800 (Beckman Coulter, USA). Moreover, FIB was measured by using a Fibrintimer II coagulometer and Multifibren U Kit (Stago, France).

C-reactive protein-to-Platelet Ratio (CPR) = C-reactive protein (mg/L)/Platelet count (10^9^/L)

Neutrophil-to-Lymphocyte*Platelet Ratio (NLPR) = Neutrophil count (10^9^/L) *100/Lymphocyte count (10^9^/L) * Platelet count (10^9^/L)

Fibrinogen-to-Platelet Ratio (FPR) = Fibrinogen (g/L) *100/Platelet count (10^9^/L)

### Statistical analysis

Continuous variables were presented as the mean ± standard deviation or as the median with interquartile ranges (IQRs). Quantitative variables with normal distribution were tested via the Student’s *t* test. Quantitative variables without normal distribution were analysed by using the Mann–Whitney *U* test. Categorical variables were described as numbers (percentages). For the categorical variables, Pearson’s chi-squared test or Fisher’s exact test was applied. Unadjusted associations between covariates and prognoses of PLA were estimated by using bivariable logistic regression models. Moreover, adjusted odds ratios (ORs) were estimated by using multivariate logistic regression models with the inclusion of covariate terms that were chosen based on these variables, including age, gender, BMI, comorbidities, imaging features, treatments and complications. Receiver operating characteristic (ROC) curves were applied to determine the optimal cut-off value, sensitivity and specificity, and areas under the ROC curves (AUCs) were calculated. Data were accepted as statistically significant when two-sided *p*-values were < 0.05. SPSS 20.0 was used for data management and statistical analyses.

## Results

A total of 372 PLA patients were included in the study. The baseline characteristics of the patients are presented in Table 1. The average age was 61.8 ± 13.5 years, and 215 patients (57.8%) were men. Diabetes mellitus (n = 155, 41.7%) was the most common underlying disease in the patients’ medical histories. Among the clinical symptoms on admission, fever (n = 323, 86.8%) was the most frequent symptom. Additionally, the most common infection site were right lobe (55.6%). *K. pneumoniae* was the main pathogen in the pyogenic liver abscess, followed by *E. coli*. Eventually, 80 patients (21.5%) developed sepsis and 33 patients (8.9%) developed septic shock. The duration of hospitalization was 14.2 ± 9.2 days. The in-hospital mortality for PLA patients was 2.4%.

**Table 1:** Baseline characteristics of patients with pyogenic liver abscess

Characteristics	Total (n = 372)
Age (years)	61.8 ± 13.5
Gender (male)	215 (57.8)
*Comorbidities*	
Hypertension	135 (36.3)
Diabetes mellitus	155 (41.7)
Fatty liver	40 (10.8)
Cholelithiasis	92 (24.7)
Viral hepatitis	57 (15.3)
Malignancy	14 (3.8)
Cardiovascular diseases	70 (18.8)
Chronic renal insufficiency	27 (7.3)
Hepatic dysfunction	70 (18.8)
Gastrointestinal surgery history*	46 (12.4)
*Clinical signs*	
Fever	323 (86.8)
Abdominal pain	162 (43.5)
Nausea	50 (13.4)
Vomiting	43 (11.6)
Frailty	62 (16.7)
Diarrhea	13 (3.5)
*Abscess location*	
Left lobe	89 (24.0)
Right lobe	207 (55.6)
Multiple lesions	76 (20.4)
*Microbiological etiology*	
*Klebsiella pneumoniae*	145 (38.9)
*Escherichia coli*	70 (18.8)
*Enterococcus spp.*	28 (7.5)
*Streptococcus spp.*	18 (4.8)
Others*	111 (29.8)
Development of the sepsis	80 (21.5)
Development of the septic shock	33 (8.9)
Hospital length of stay, days	14.2 ± 9.2
ICU admission	29 (7.8)
Metastatic infection	20 (5.4)
In-hospital mortality (%)	9 (2.4)

^*^Gastrointestinal surgery history in the past six months

^*^Others: *Pseudomonas aeruginosa*, *Acinetobacter baumannii*, *Enterobacter aerogenes*, *Proteus mirabilis*, *Citrobacter spp.*, *Staphylococcus spp.*, *Anaerobes*, culture-negative

As shown in Table 2, patients with sepsis had more incidences of fatty liver, chronic renal insufficiency and hepatic dysfunction than the non-sepsis group. In terms of microbiological etiology, the sepsis group had a significantly higher proportion of *K. pneumoniae* than the non-sepsis group. The patients who were hospitalized for more than 21 days showed higher rates of hypertension, diabetes mellitus, fatty liver, chronic renal insufficiency, cardiovascular diseases and hepatic dysfunction (*p* < 0.05 for all factors). In addition, the patients with prolonged hospital stays were significantly older and had a higher rate of large liver abscesses (> 10 cm). More frequent development of complications including acute respiratory distress syndrome, spontaneous rupture of abscess, and extrahepatic manifestations was found in the sepsis group and prolonged hospital stays group.

**Table 2:** Demographic characteristics and laboratory findings of pyogenic liver abscess patients with sepsis or prolonged hospital stays

	Sepsis			Prolonged hospital stays		
	Sepsis (n = 80)	Non-sepsis (n = 292)	*p* value	> 21d (n = 61)	≤ 21d (n = 311)	*p* value
Age (years)	63.5 ± 13.6	61.4 ± 13.4	0.210	65.8 ± 13.8	61.0 ± 13.3	**0.011**
Gender (male), n (%)	50(62.5)	165(56.5)	0.336	30(49.2)	127(40.8)	0.228
BMI	24(22–26)	23.5(22–26)	0.115	23.5(21.3–25.5)	24(22–26)	0.717
Comorbidities						
Hypertension	31(38.8)	104(35.6)	0.606	30(49.2)	105(33.8)	**0.022**
Diabetes mellitus	39(48.8)	116(39.7)	0.147	33(54.1)	122(39.2)	**0.031**
Fatty liver	16(20.0)	24(8.2)	**0.003**	12(19.7)	28(9.0)	**0.014**
Cholelithiasis	16(20.0)	76(26.0)	0.268	16(26.2)	76(24.4)	0.767
Viral hepatitis	9(11.2)	48(16.4)	0.254	8(13.1)	49(15.8)	0.601
Chronic renal insufficiency	20(25.0)	7(2.4)	** < 0.001**	12(19.7)	15(4.8)	** < 0.001**
Malignancy	5(6.2)	9(3.1)	0.187	3(4.9)	11(3.5)	0.604
Cardiovascular diseases	16(20.0)	54(18.5)	0.760	18(29.5)	52(16.7)	**0.019**
Hepatic dysfunction	30(37.5)	40(13.7)	** < 0.001**	19(31.1)	51(16.4)	**0.007**
Gastrointestinal surgery history	8(10.0)	38(13.0)	0.468	6(9.8)	40(12.9)	0.512
Microbiological etiology			**0.022**			0.173
*Klebsiella pneumoniae*	39(48.8)	106(36.3)		30(49.2)	115(37.0)	
*Escherichia coli*	18(22.5)	52(17.8)		11(18.0)	59(19.0)	
*Others*	23(28.8)	134(45.9)		20(32.8)	137(44.1)	
**Abscess size(cm)**			0.994			**0.025**
< 5	21(26.2)	75(25.7)		11(18.0)	85(27.3)	
5–10	54(67.5)	199(68.2)		42(68.9)	211(67.8)	
> 10	5(6.2)	18(6.2)		8(13.1)	15(4.8)	
**Abscess number**			0.914			0.612
Single lesion	64(80.0)	232(79.5)		50(82.0)	246(79.1)	
Multiple lesions	16(20.0)	60(20.5)		11(18.0)	65(20.9)	
**Treatment**			0.168			0.060
Antibiotics alone	14(17.5)	78(26.7)		8(13.1)	84(27.0)	
Antibiotics + Percutaneous drainage	62(77.5)	206(70,5)		50(82.0)	218(70.1)	
Antibiotics + Surgical drainage	4(5.0)	8(2.7)		3(4.9)	9(2.9)	
**Complications**						
Acute respiratory distress syndrome	6(7.5)	2(0.7)	**0.000**	5(8.2)	3(1.0)	**0.004**
Peritoneal effusion	11(13.8)	22(7.5)	0.083	8(13.1)	25(8.0)	0.202
Spontaneous rupture of abscess	4(5.0)	1(0.3)	**0.008**	3(4.9)	2(0.6)	**0.033**
Extrahepatic manifestations	16(20.0)	4(1.4)	**0.000**	10(16.4)	10(3.2)	**0.000**
ICU admission	22(27.5)	7(2.4)	0.000	14(23.0)	15(4.8)	**0.000**
Laboratory findings						
WBC (10^9^/L)	12.5(10.6–16.8)	11.2(8.8–15.2)	**0.013**	12.5(10.5–16.6)	11.3(8.9–15.2)	**0.047**
Hb (g/L)	120.0(108.0–133.8)	118.5(105.0–129.0)	0.316	120(101.5–128)	119(106–130.5)	0.459
PLT (10^9^/L)	98.0(66.0–167.5)	221.5(151.5–332.8)	** < 0.001**	135(79.5–225)	210(136–318)	** < 0.001**
Neu (10^9^/L)	11.1(9.2–15.1)	9.1(6.9–12.6)	** < 0.001**	10.7(9.0–14.5)	9.4(7.0–12.9)	**0.007**
Ly (10^9^/L)	0.6(0.5–1.0)	1.2(0.7–1.6)	** < 0.001**	0.7(0.5–1.3)	1.1(0.7–1.5)	**0.003**
NLR	19.2(10.4–26.5)	8.3(5.1–14.7)	** < 0.001**	15.3(8.8–21.5)	9.3(5.2–15.9)	** < 0.001**
BUN (mmol/L)	8.2(5.2–12.4)	4.6(3.6–6.0)	** < 0.001**	6.9(4.2–10.2)	4.8(3.7–6.5)	** < 0.001**
Cr (μmol/L)	78.0(63.5–110.3)	63.0(53.0–78.0)	** < 0.001**	76(51–100.5)	66(54–78.5)	0.050
ALT (U/L)	71.0(43.5–115.5)	45.0(26.0–77.0)	** < 0.001**	56(35.5–104.5)	48(27–82.5)	0.148
ALP (U/L)	152.5(104.0–234.0)	166.0(117.8–229.8)	0.219	174(105.5–222.5)	162(116–233.5)	0.779
TBIL (μmol/L)	19.7(13.0–37.4)	14.0(9.4–21.1)	** < 0.001**	16.7(10.6–29.1)	14.3(9.8–23.9)	0.145
TBA (μmol/L)	8.2(4.3–20.0)	5.2(2.9–9.9)	** < 0.001**	9.4(5–15.5)	5.3(2.8–10.1)	**0.002**
ALB (g/L)	28.5 ± 5.6	31.6 ± 5.6	** < 0.001**	28.8 ± 4.7	31.4 ± 5.8	** < 0.001**
CRP (mg/L)	188(140.9–266.1)	114(50.1–173)	** < 0.001**	176.7(141.2–239.9)	120(50.1–183.0)	** < 0.001**
FIB (g/L)	6.7(5.7–7.8)	6.4(5.6–7.6)	0.302	6.9(6.0–7.9)	6.4(5.6–7.6)	**0.044**
CPR	1.8(1.0–3.6)	0.5(0.2–1.0)	** < 0.001**	1.5(0.9–3.0)	0.5(0.3–1.2)	** < 0.001**
NLPR	16.8(8.6–33.2)	3.6(1.6–9.7)	** < 0.001**	13.4(5.2–26.6)	4.6(1.8–12.0)	** < 0.001**
FPR	6.01(3.8–11.4)	2.8(1.9–4.2)	** < 0.001**	5.1(2.8–10.1)	3.0(2.0–4.8)	** < 0.001**

Bold type indicates statistical significance (*p* < 0.05)

*WBC* white blood cells, *Hb* hemoglobin, *PLT* platelets, *Neu* neutrophils, *Ly* lymphocytes, *NLR* neutrophil to lymphocyte, *BUN* blood urea nitrogen, *Cr* creatinine, *ALT* alanine transaminase, *ALP* alkaline phosphatase, *TBIL* total bilirubin, *TBA* total bile acid, *ALB* albumin, *CRP* C-reactive protein, *FIB* fibrinogen, *CPR* C-reactive protein-to-platelet ratio, *NLPR* neutrophil-to-lymphocyte*platelet ratio, *FPR* fibrinogen-to-platelet ratio

We compared the laboratory parameters among the different groups of PLA patients. In the sepsis group, white blood cells (WBC), neutrophils (Neu), neutrophil-to-lymphocyte ratio (NLR), blood urea nitrogen (BUN), creatinine (Cr), alanine transaminase (ALT), total bilirubin (TBIL), total bile acid (TBA), CPR, NLPR and FPR were significantly higher than those in the non-sepsis group. In contrast, patients with sepsis had significantly lower PLT, lymphocyte (Ly), alkaline phosphatase (ALP), albumin (ALB) levels than patients with non-sepsis (*p* < 0.05, all of them). Patients with prolonged hospital stays had significantly higher WBC, Neu, NLR, BUN, TBA, CPR, NLPR and FPR levels, as well as lower PLT, Ly and ALB levels than those patients without prolonged hospital stays (Table 2; Additional file 1).

A multivariate regression analysis was used, and parameters including age, gender, BMI, comorbidities, microbiological etiology, imaging features, treatments and complications were included. The multivariate analysis showed that CPR (OR: 2.262, 95% CI: 1.586–3.226, *p* < 0.001), NLPR (OR: 1.118, 95% CI: 1.070–1.167, *p* < 0.001) and FPR (OR: 1.197, 95% CI: 1.079–1.329, *p* = 0.001) were independent risk factors for PLA associated with sepsis (Table 3). In addition, the results of the analysis demonstrated that NLPR (OR: 1.019, 95% CI: 1.004–1.046, *p* = 0.019) was an important indicator closely related to prolonged hospital stays.

**Table 3:** Univariable and multivariate analysis for PLA patients with sepsis or prolonged hospital stays

Baseline variables	Sepsis				Prolonged hospital stays			
	Unadjusted OR (95%CI)	*p* value	Adjusted OR (95%CI) *	*p* value	Unadjusted OR (95%CI)	*p* value	Adjusted OR (95%CI) *	*p* value
*Model 1*								
CPR	2.731(2.052–3.634)	** < 0.001**	2.262(1.586–3.226)	** < 0.001**	1.030(0.992–1.071)	0.126	–	–
*Model 2*								
NLPR	1.097(1.068–1.127)	** < 0.001**	1.118(1.070–1.167)	** < 0.001**	1.016(1.006–1.026)	**0.002**	1.019(1.004–1.046)	**0.019**
*Model 3*								
FPR	1.288(1.183–1.401)	** < 0.001**	1.197(1.079–1.329)	**0.001**	1.017(0.997–1.037)	0.095	–	–

Bold type indicates statistical significance (*p* < 0.05)

^*^Age, gender, BMI, comorbidities, microbiological etiology, abscess size, abscess number, treatments and complications were included in multivariate analysis

**Fig. 1:**
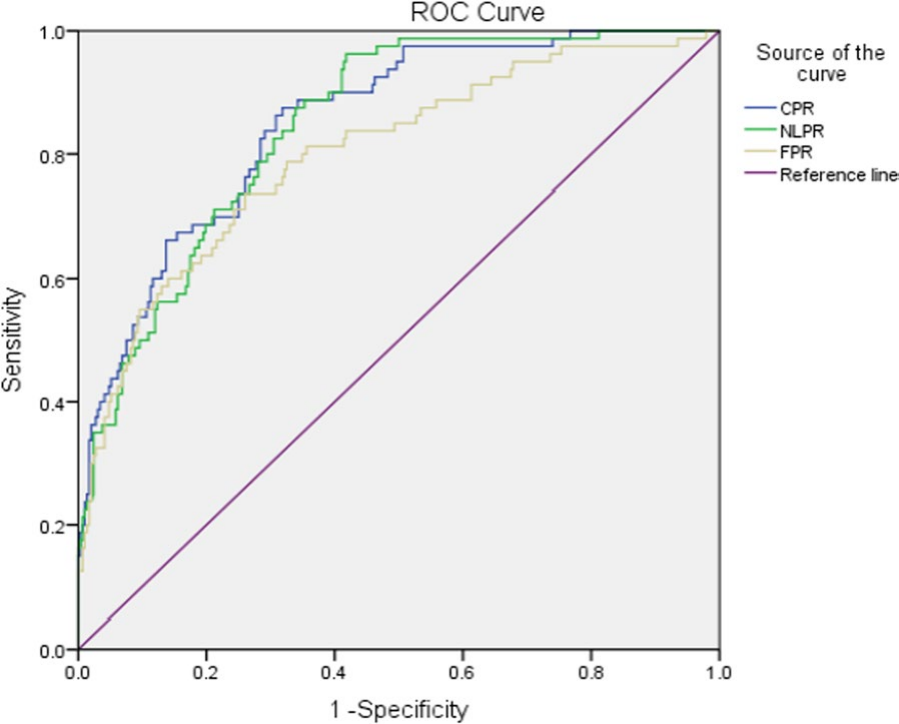
ROC curve of CPR, NLPR and FPR in the differential diagnosis of pyogenic liver abscess patients with sepsis

**Fig. 2:**
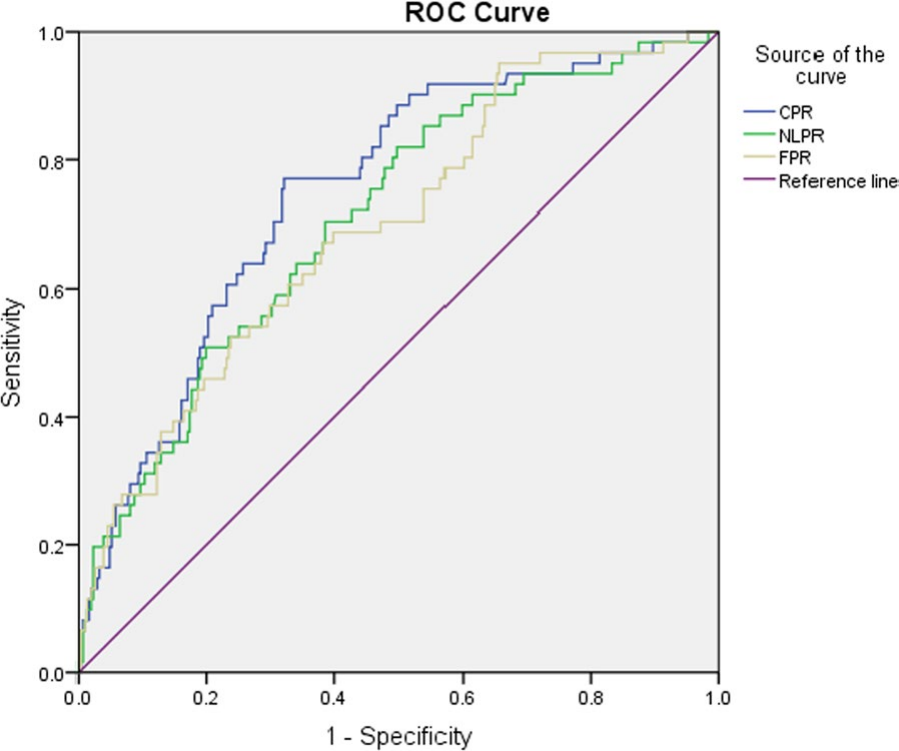
ROC curve of CPR, NLPR and FPR in the differential diagnosis of pyogenic liver abscess patients with prolonged hospital stays

In the ROC curve analysis, the area under the curve was 0.851 (95% CI: 0.807–0.896) for CPR, 0.847(95% CI:0.804–0.889) for NLPR and 0.801(95% CI:0.743–0.858) for FRP, between the patients with and without sepsis (Fig. 1 and Table 4). The comparison between the prolonged hospital stays group and those without group yielded AUCs for CPR, NLPR and FPR of 0.750, 0.710 and 0.696, respectively (Fig. 2 and Table 4).

**Table 4:** Diagnostic value of CPR, NLPR and FPR for PLA patients with sepsis and prolonged hospital stays

Biomarker	AUC (95%CI)	Cut-off value	Sensitivity (%)	Specificity (%)	*p* value
*Sepsis*					
CPR	0.851(0.807–0.896)	0.8	87.5	68.2	** < 0.001**
NLPR	0.847(0.804–0.889)	5.9	88.8	64.7	** < 0.001**
FPR	0.801(0.743–0.858)	4.1	73.8	74.0	** < 0.001**
*Prolonged hospital stays*					
CPR	0.750(0.686–0.814)	0.9	77.0	67.8	** < 0.001**
NLPR	0.710(0.640–0.779)	4.6	82.0	50.2	** < 0.001**
FPR	0.696(0.625–0.766)	3.5	68.9	60.1	** < 0.001**

Bold type indicates statistical significance (*p* < 0.05)

## Discussion

Currently, pyogenic liver abscess remains a rare infectious disease. PLA is caused by ascending bacteria that spread via the biliary tract or systemic bloodstream infections [20, 21], which is complicated by sepsis, septic shock and metastatic infections [3]. Thus far, the most useful biomarker for predicting pyogenic liver abscess has not been established. Therefore, there is a need for more sensitive and lower-cost predictors, especially in rural areas of developing countries. However, little is known about comparing the prognostic value of combining platelets and inflammatory biomarkers in patients with PLA.

In our present study, the 372 PLA patients were divided into two different groups, sepsis and non-sepsis, as well as hospitalizations for more than 21 days (≥ 21 days) and hospitalizations for less than 21 days (< 21 days), according to complications and the clinical outcome. Patients with prolonged hospital stays had significantly more underlying conditions. Moreover, the sepsis group had more serious infections, lower platelet counts, worse renal function and liver function. Moreover, septic patients were associated with dysregulated immune defences and complex microbiome-host interactions.

Serum C-reactive protein, neutrophil count, lymphocyte count and fibrinogen are convenient accessible biomarkers that can be measured in the blood. C-reactive protein is an acute-phase protein synthesized by the liver and associated with the inflammatory situations of the body. Foo et al. found that C-reactive protein was a good marker of PLA [18]. The availability of CRP in predicting the recovery and adequacy of antibiotic therapy has been previously investigated. Weekly CRP measurements were found to be helpful in the prediction of PLA patients with good outcomes [8]. As a simple non-specific marker of inflammation, NLR is a reliable predictor of systemic inflammation and involved in the development and progression of the disease. Neutrophils are one of earliest inflammatory responders of cells to defend the body against the invasion of pathogens [22]. Contrary to neutrophils, lymphocytes provide a broader and more finely tuned repertoire of recognition for antigens [23]. Foo et al. demonstrated that approximately 90% of PLA patients had increased neutrophil counts (neutrophil count > 70%) [18]. In addition, Kong et al. showed that the delta neutrophil index (DNI) predicted the development of shock or in-hospital hypotension in patients with PLA [24]. A previous study by Park et al. showed that NLR was strongly correlated with poor prognoses of PLA in the emergency department, and increased NLR implied a high risk of death, ICU admission, and development of septic shock [25]. In this study, we found that C-reactive protein and NLR were increased in the sepsis and prolonged hospital stays group, thus indicating that C-reactive protein and NLR may play essential roles in the prognosis of PLA.

The results of our study confirmed that there was a remarkable decline in the PLT level. In the infectious diseases, platelets are at the crossroads of the immune response and coagulation [26]. Bacteria can either assist in platelet adhesion or promote platelet aggregation [27]. In the initial phase of inflammation, activated platelets can capture neutrophils and interact with neutrophils to exacerbate the systemic inflammatory response [28, 29]. Platelets are considered to play a crucial role in vascular inflammation and can be rapidly depleted when vessels are in the acute inflammation stage [30]. Platelet consumption and/or the relative decrease in the free platelet proportion can result in lower platelet levels [31]. Research has shown that PLTs are involved in the hepatic response to the initial phase of sepsis, and platelet accumulation can contribute to liver dysfunction [32]. Reduced platelets have also been found to be associated with a poor prognosis in sepsis patients [33].

The combination of platelets and inflammatory biomarkers has been demonstrated to be associated with poor prognoses in a variety of clinical situations, including sepsis and cardiovascular diseases [34–36]. Li X found that a higher CPR was an independent risk factor for neonatal sepsis [11]. In addition, a previous study demonstrated that NLPR was associated with early acute kidney injury in cardiovascular surgery [31]. In the present study, we innovatively combined platelets with traditional inflammatory indicators. Our results showed that the CPR, NLPR and FPR were independent risks for developing sepsis in PLA patients after adjustment for age, gender, BMI, comorbidities, microbiological etiology, abscess size, abscess number, treatment and complications. Furthermore, NLPR was an independent risk factor for prolonged hospital stays. For the development of sepsis, the area under the curve of CPR was 0.851, compared to 0.847 for NLPR and 0.801 for FPR. In this study, CPR had the best effect on early prediction for sepsis, which was superior to FRP and not inferior to NLPR. CPR had a good predictive value for sepsis with a sensitivity of 87.5% and a specificity of 68.2%. We speculated that CPR may be better used to assess the status of hepatic metabolism and coagulation. The possible mechanisms involved with these new indicators need to be further investigated.

Our study had several limitations. First, it was a single center study, which may have resulted in bias. The conclusion still requires further validation with a larger sample size from multiple-centers. Second, the hospital mortality of PLA patients was low. Therefore, our study did not include mortality as a prognostic factor. Third, data on the time of treatment initiation and the delay of the drainage procedure were not recorded, and we were unable to consider it as a risk factor in the analysis. Finally, we only measured the serum markers within the first 24 h after hospital admission, and these markers may change dynamically during different stages of the disease. The monitoring of the dynamic changes in these inflammatory indicator levels and combining them with other biomarkers may be important for further research.

In summary, CPR, NLPR and FPR levels on admission were correlated with sepsis in PLA patients as well as prolonged hospital stays. Furthermore, CPR can be recommended to have superior prognostic value in pyogenic liver abscesses compared with NLPR and FPR.

## Supplementary Information

**Additional file 1.** The original data presented in the study are included in the Supplementary Information.

## Data Availability

The datasets analyzed during the current study are available from the corresponding author on reasonable request.
